# Electrophysiological Correlates of Change Detection during Delayed Matching Task: A Comparison of Different References

**DOI:** 10.3389/fnins.2017.00527

**Published:** 2017-09-26

**Authors:** Tengfei Liang, Zhonghua Hu, Yuchen Li, Chaoxiong Ye, Qiang Liu

**Affiliations:** ^1^Research Center of Brain and Cognitive Neuroscience, Liaoning Normal University, Dalian, China; ^2^Department of Psychology, University of Jyväskylä, Jyväskylä, Finland; ^3^Faculty of Information Technology, University of Jyväskylä, Jyväskylä, Finland

**Keywords:** REST reference, average reference, linked mastoid, change detection, N270, P2, delayed matching task

## Abstract

Detecting the changed information between memory representation and incoming sensory inputs is a fundamental cognitive ability. By offering the promise of excellent temporal resolution, event-related potential (ERP) technique has served as a primary tool for studying this process with reference of the linked mastoid (LM). However, given that LM may distort the ERP signals, it is still undetermined whether LM is the best reference choice. The goal of the current study was to systematically compare LM, reference electrode standardization technique (REST) and average reference (AR) for assessing the ERP correlates of change detection during a delayed matching task. Colored shapes were adopted as materials while both the task-relevant shape feature and -irrelevant color feature could be changed. The results of the ERP amplitude showed that both of the task-relevant and -conjunction feature changes elicited significantly more positive posterior P2 in REST and AR, but not in LM. Besides, significantly increased N270 was observed in task-relevant and -conjunction feature changes in both the REST and LM, but in the conjunction feature change in AR. Only the REST-obtained N270 revealed a significant increment in task-irrelevant feature change, which was compatible with the delayed behavioral performance. Statistical parametric scalp mapping (SPSM) results showed a left posterior distribution for AR, an anterior distribution for LM, and both the anterior and left posterior distributions for REST. These results indicate that different types of references may provide distinct cognitive interpretations. Interestingly, only the SPSM of REST was consistent with previous fMRI findings. Combined with the evidence of simulation studies and the current observations, we take the REST-based results as the objective one, and recommend using REST technology in the future ERP data analysis.

## Introduction

The detection of changed information between memory representation and perceptual inputs is of considerable importance to our cognitive process. It helps adjust the current cognitive operation to promote appropriate behaviors in keeping with the ever-changing environments (Hollingworth et al., 2008; Richard et al., 2008). Previous studies of this field generally adopt the delayed matching paradigm. In this task, a memory item is displayed and followed by a test item some 100 ms later that is either change or no change from the foregoing memory item. By exploiting this paradigm, Cui et al. (2000) found that the change detection processing could be tracked by a frontal distributed N270 component. After them, this component was confirmed by the delayed matching task with various stimulus materials, like the number amplitude (Li et al., 2003), cross-modal gender mismatch (Wang et al., 2002) and the arithmetic conflicts (Newman and Connolly, 2004). N270 was also used in clinical studies to assess the patient's cognitive function (Wang et al., 2002; Sun et al., 2008). Cortical source localization evidences showed that N270 may generate from the anterior cingulate cortex (ACC) (Yin et al., 2012). In consideration of a strong connectivity existing between ACC and the dorsolateral prefrontal cortex (DLPFC) (Wang et al., 2010), it was proposed that N270 reflects the downstream process (Zhang et al., 2008; Scannella et al., 2016). This mechanism is based upon the afferent ACC signals and takes place within the DLPFC to generate an appropriate behavior response (Scannella et al., 2016).

Not only the anterior region, the posterior area was also found to be involved in the processing of change detection in delayed matching task, especially the left occipito-temporal cortex (Zhang et al., 2008). However, no corresponding electrophysiological signal was found from this brain area. What needs to be pointed out is that previous studies focused on this process mainly used the linked mastoid (LM) as the reference (Cui et al., 2000; Wang et al., 2002; Li et al., 2003; Sun et al., 2008; Scannella et al., 2016), which is the average of the left and right mastoids. While Yao et al. (2005) found that LM-obtained EEG power was markedly shifted to the frontal and superficial positions in an unreasonable way. Based on such observation, it is reasonable to speculate that the improper use of the LM reference may weaken the strength of posterior electrophysiological signals, thus makes the posterior task related ERP effects disappeared. In fact, not just the scalp distribution of power spectra, the wave amplitude of the scalp potentials, large-scale networks and non-linear features of EEG were also shown to be distorted by the use of LM (Kayser et al., 2007; Marzetti et al., 2007; Yao et al., 2007; Qin et al., 2010; Tian and Yao, 2013; Chella et al., 2017; Lei and Liao, 2017; Yang et al., 2017). Besides, taking into account the fact that there is no neutral location exists on the human body surface (Nunez et al., 1997), the average reference (AR) that relying on human upper scalp electrodes also faces similar theoretical defect (Yao, 2017). Fortunately, a newly reference electrode standardization technique (REST) was provided to compensate for this problem (Yao, 2001). Unlike the channel-based reference methods, such as AR and LM, the REST technique can avoid the use of the human body surface positions as reference by establishing a point at infinity to constitute a neutral reference. The superiority of the REST has also been confirmed by several lines of evidences. For instance, by investigating the effects of reference choice on the analysis of the non-linear features of the EEG signals, Chella et al. (2017) found that compared with all other types of references (with vertex electrode, LM AR and REST in their study), the REST technology can do a better job in approximating the zero value. With less error, Lei and Liao (2017) also observed that REST could be adopted as the preferable reference for all large-scale networks, regardless of the EEG signal-to-noise ratios and the electrodes number. Based on these findings, it is reasonable to predict that some new insights would also be got in the electrophysiological correlates of change detection if the REST technology is adopted.

To test this hypothesis, EEG data in delayed matching task were referenced to LM, AR, and REST methods with the ERP amplitude, voltage scalp distribution and the signals statistical parametric scalp mapping (SPSM) were measured. In order to create a change circumstance between memory representation and perceptual input, interval in delayed matching task was set to 1,000 ms. Given that the iconic memory trace of one item could only be last about 250 ms (Gegenfurtner and Sperling, 1993), it is sufficient to ensure that the first item was stored into visual working memory. We were also concerned with whether the REST is a more sensitive reference technique than the other two types of methods. To this end, colored shapes were adopted and its change types were manipulated by equally changing the task-relevant shape feature and -irrelevant color feature. Based on this operation, we aimed at investigating the sensitivity of different types of references to the N270 component elicited by the task-irrelevant feature change.

## Materials and methods

### Participants

All participants were recruited from the Liaoning Normal University community and reported normal or corrected-to-normal vision and none had a history of neurological disorders. Fourteen right-handed students (10 males) with a mean age of 22.5 years (range 20–26) participated in this study. This experiment was carried out in accordance with the recommendations of procedures and protocols approved by the human subjects review committee of Liaoning Normal University with written informed consent from all participants.

### Stimuli

Stimuli were colored shape (2.38° × 2.38° images, 70 cm in front of the participants). The color of stimuli was randomly selected from a set of five colors: green (0, 255, 0), red (255, 0, 0), yellow (255, 255, 0), blue (0, 0, 255), and violet (255, 0, 255), and their shapes were randomly selected from a set of five shapes (crisscross, round, triangle, square and heart). Stimuli were presented on a 17-inch CRT monitor (1,024 × 768 pixels, 85 Hz refresh-rate), with a gray background (128, 128, 128).

### Experimental procedures

**Figure 1:**
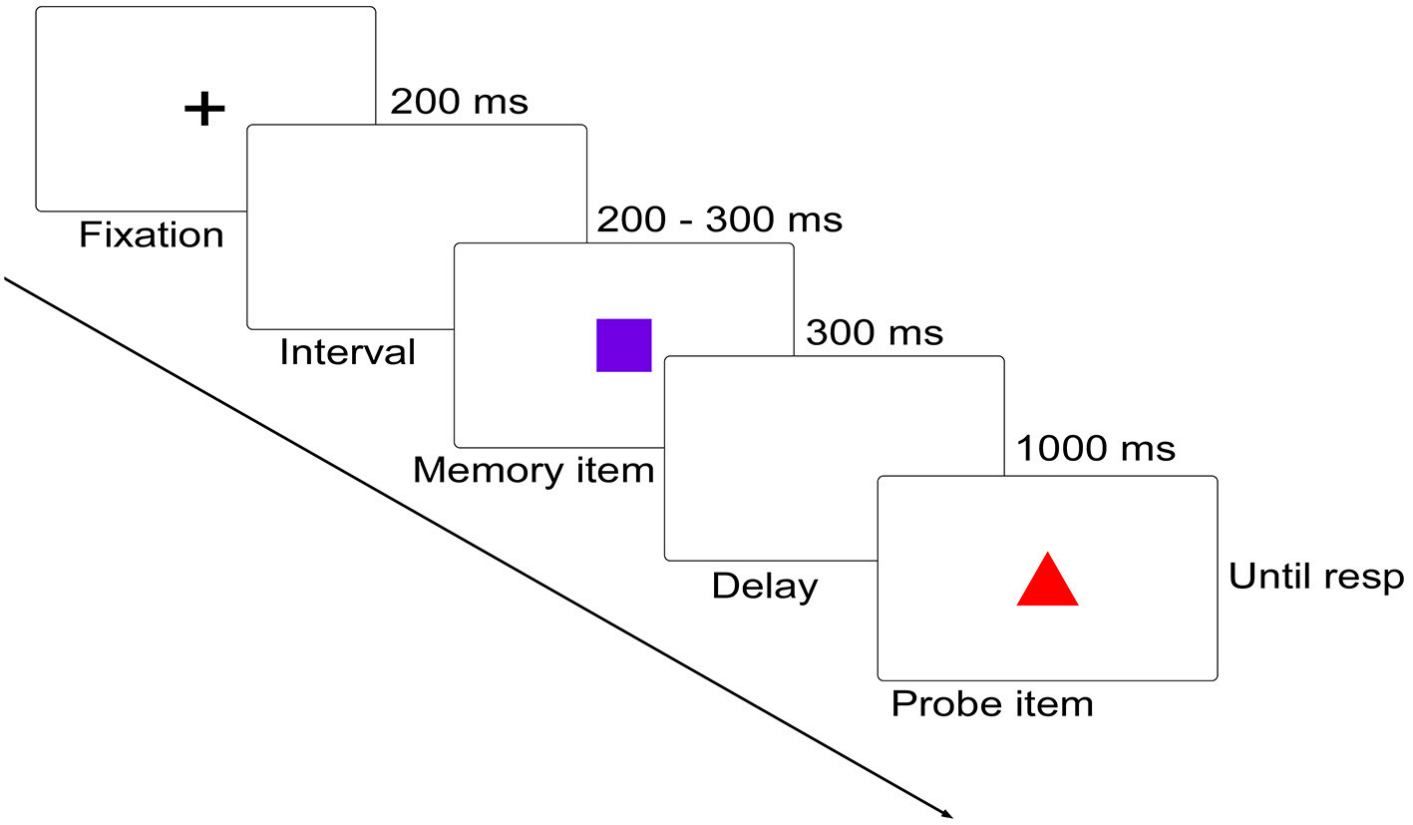
Schematic diagram (not to scale) of the delayed matching task. In this diagram, both of the shape (relevant) and color (irrelevant) features were changed. Below are the five distinct shapes and colors used in the current study.

Participants performed a delayed matching task in which they had to detect the change between two successively presented figures. On each trial, memory item was presented for 300 ms, followed by a 1,000 ms blank display, and then by a probe display, which remained until participants responded using the keyboard (Figure 1). Participants were required to remember the shape feature of the memory item while ignoring its color feature, and judge whether the shape of the probe item was as same as that of the memory item. Participants were informed of pressing “F” on the keyboard for shape feature changed trials and “J” for no shape feature changed trials as quickly and accurately as possible. Once the responses were initiated, a 1,000–1,400 ms blank display would be presented before the start of the next trial.

Four interested conditions were included: (1) memory item and probe item were complete same (No change, NC); (2) probe item was different from the memory item in the task-irrelevant color feature (Irrelevant-change, IC); (3) probe item was different from the memory item in the task-relevant shape feature (Relevant-change, RC); (4) both of the items were different in their color feature and shape feature (Conjunction-change, CC). There were 10 practice trials and 400 experimental trials, with 100 trials for each condition. Besides, another visual task was also invited to complete. Since this task has nothing to do with the current study, the data were not included in the current report.

### EEG recording

The EEG signals were recorded by using a 64-channel amplifier ANT Neuro EEGO mounted in a cap using 10/20 montage. Horizontal and vertical EOG were recorded bipolar from the outer canthi of the eyes and from above and below the observer's left eye. The GND electrode served as the ground electrode and CPz served as the on-line reference. Electrode impedances were kept below 10 kV with a sampling rate at 500 Hz for off-line analysis.

### EEG data preprocess and re-reference

EEGLAB (Makeig et al., 2004) and Letswave (Mouraux and Iannetti, 2008) were used for off-line processing. Original EEG signals were digitally filtered with the band-pass of 0.1 and 30 Hz. EEG epochs were then extracted with a time window of 2,000 ms (−500 ms pre-stimulus and 1,500 ms post-stimulus) for independent component decomposition. Before independent component analysis algorithm (Makeig et al., 2004) was adopted to correct EOG artifacts, trials corresponding to error or contaminated by gross movements were removed manually. After the independent component analysis, epochs were re-segmented into a time window of 1,200 ms (−200 ms before the probe item and 1,000 ms after the probe item) for ERP analysis. Baseline correction was performed using the pre-stimulus interval (−200 to 0 ms before the probe item).

After that, the remaining EEG trials were referenced to AR, LM, and REST references. LM used the average of the bilateral mastoids as reference. AR reference used the average of all channels as reference. Whereas REST (Yao, 2001) was conducted by the REST software from www.neuro.uestc.edu.cn/rest. Notably, re-reference was done after all the preprocessing had been completed. This measure could effectively avoid the impact of distinctive artifact removal standards on the reference effects.

**Figure 2:**
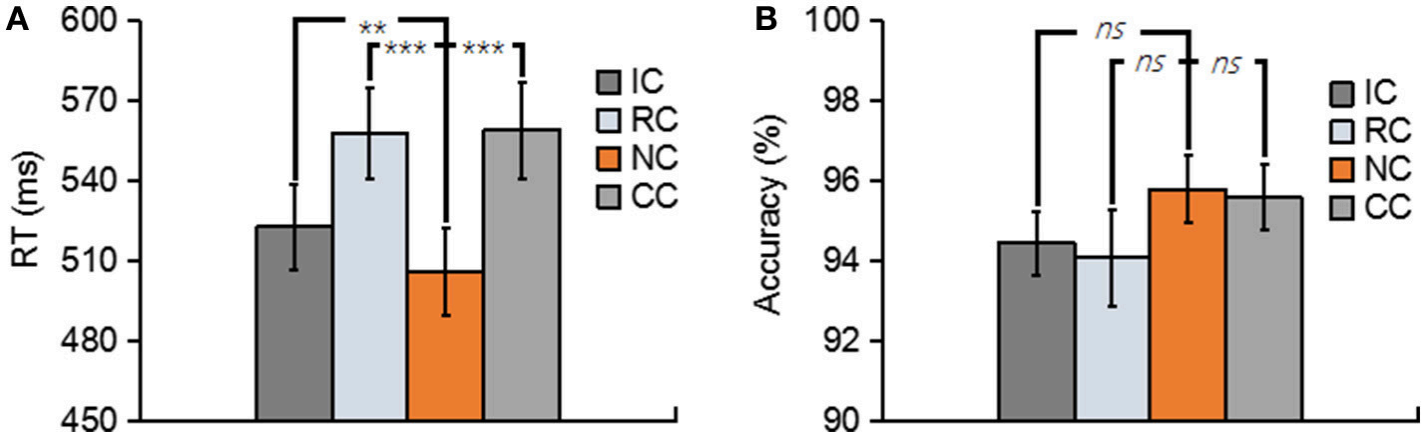
**(A)** Depicts the mean reaction time (RT) for No change (NC), Irrelevant-change (IC), Relevant-change (RC), and Conjunction-change (CC) conditions. **(B)** Depicts the mean accuracy for four conditions. Error bars represent the standard errors. ns, non-significant; ^**^p < 0.01; ^***^p < 0.001.

For all the data in the present study, only the correct-response trials were analyzed. We adopted an exploratory data-driven analysis to identify the time regions of interest and their corresponding spatial regions of interest, which were the most significantly modulated by the factor of change type. Before proceeding the exploratory data-driven analysis, it is necessary to point out that we were only interested in the time window of 600 ms after probe item onset, for the reason that participants tended to finish their responses within about 600 ms after the memory item onset (see Figure 2A). To do the exploratory data-driven analysis, each time point of the waveforms was compared using a point-by-point one-way repeated-measure ANOVA, with change types (IC. RC, NC and CC) as the factor, combined with a non-parametric cluster-based permutation test (Maris and Oostenveld, 2007). This procedure was repeated 5,000 times. It yielded a data-driven distribution of a time map of *F*-value and *p*-value. Time points with a *p*-value ≤ 0.01 were selected to identify the time regions of interest and their corresponding spatial regions of interest. Scalp distributions of the *p*-value were used to form the statistical parametric scalp mapping (SPSM), which was used as the experimental effects of each reference.

Then, the mean amplitudes within the time regions of interest of the N270 and posterior P2 at the corresponding spatial regions of interest for each experimental condition were calculated. These mean amplitude values were then entered into a one-way repeated-measure ANOVA with the factor of change type. Greenhouse Geisser Epsilon was carried out to adjust the degrees of freedom if necessary. Significant main effect was followed by *post-hoc* contrasts with the Bonferroni correction.

## Results

### Behavioral results

Trials with a response time (RT) of less than 200 ms or greater than two standard deviations from the mean RT were removed. both the RT and the accuracy for each type of experimental condition are shown in Figure 2. In terms of RT, a one-way repeated-measures ANOVA with the factor of change type revealed a significant main effect [*F*_(3, 39)_ = 29.76, *p* < 0.01, $\eta_{p}^{2}$ = 0.70] (Figure 2A). Planned comparison contrast showed that the RC (*p* < 0.01), IC (*p* < 0.01) and CC (*p* < 0.01) significantly lengthened the response time, compared with NC. RC (*p* < 0.01) and CC (*p* < 0.01) also significantly lengthened the response time when compared with IC. The other ones were non-significant [all *p* > 0.5]. The above results suggested that whether the changed features were task relevant or not, they all considerably influenced the performance of the participants, thus generated a robust change conflict.

An identical ANOVA on the accuracy data was conducted. As shown in Figure 2B, accuracies were near ceiling at all conditions with all the accuracy data were greater than 94%, and no significant difference was found among four conditions [*F*_(3, 39)_ = 1.44, *p* = 0.25].

### ERP results

**Figure 3:**
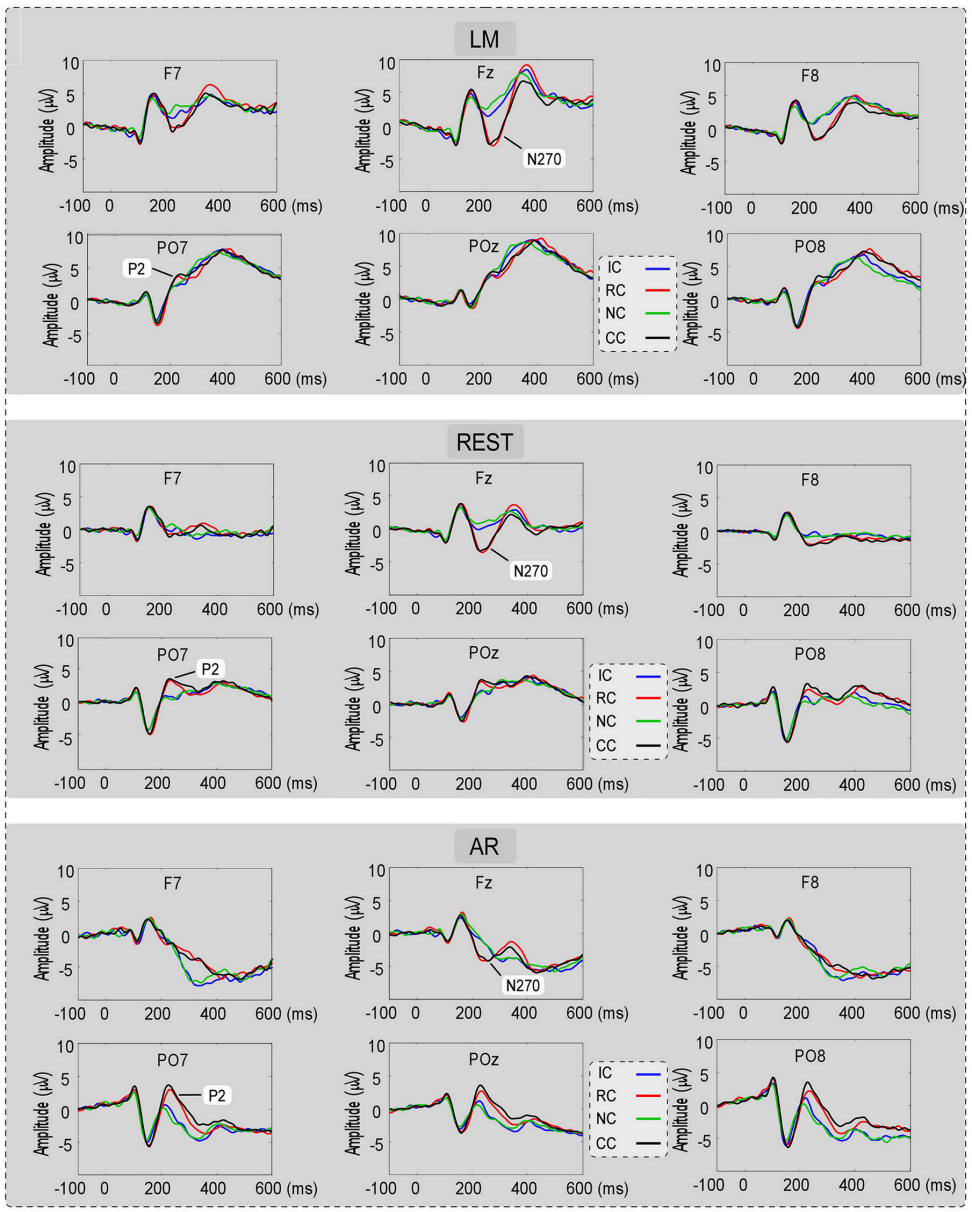
The ERP waveforms for No change (NC), Irrelevant-change (IC), Relevant-change (RC), and Conjunction-change (CC) conditions in six electrodes (F7, FZ, F8, PO7, POz, and PO8), for three references separately.

**Figure 4:**
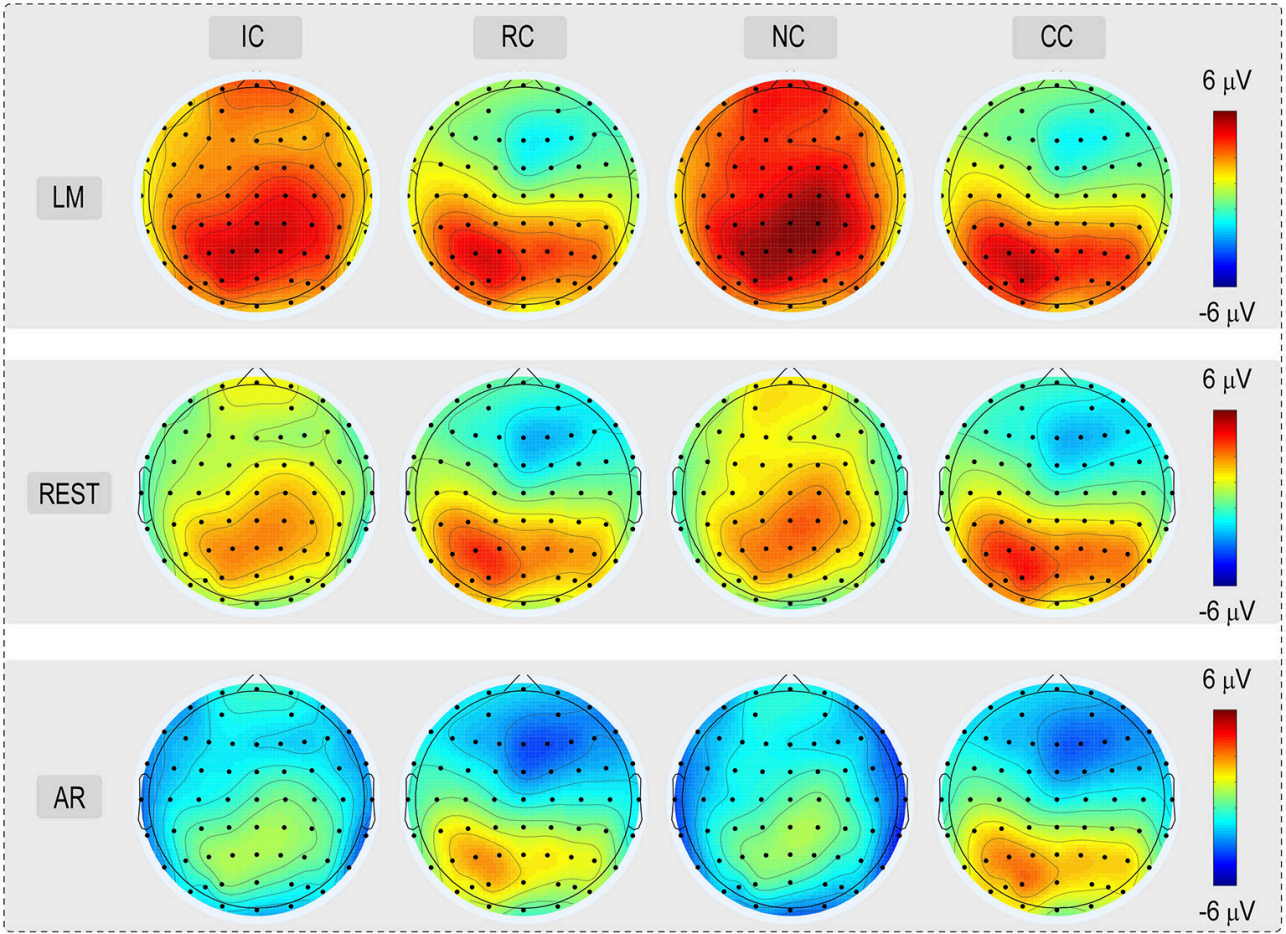
Voltage topographies (210–290 ms) of posterior P2 and N270 for No change (NC), Irrelevant-change (IC), Relevant-change (RC), and Conjunction-change (CC) conditions, for three references separately.

**Figure 5:**
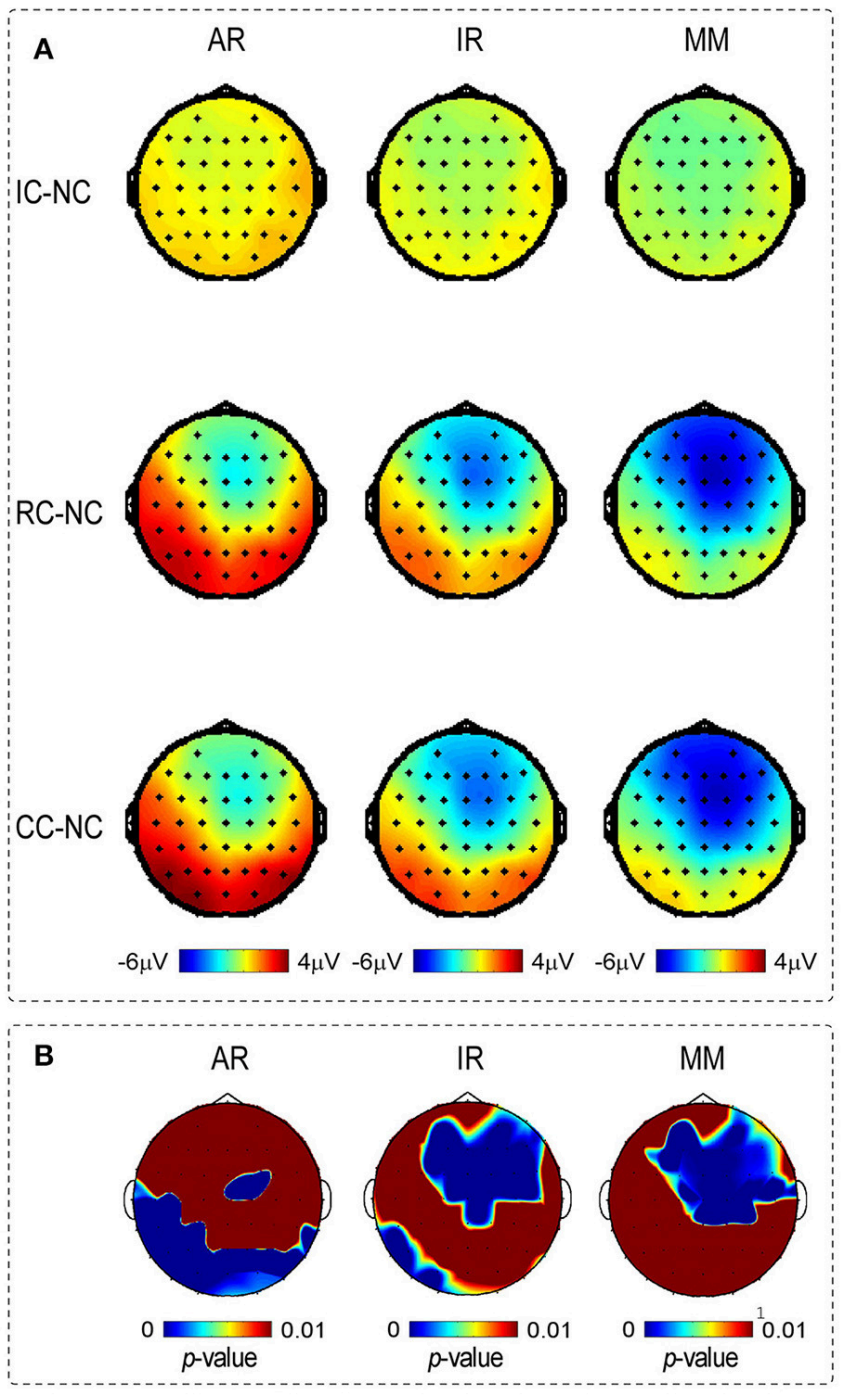
**(A)** Depicts the voltage topographies (210–290 ms) of the different waveforms of posterior P2 and N270, for three references separately. Different waveforms were calculated by subtracting the Irrelevant-change (IC), Relevant-change (RC) and Conjunction-change (CC) from No change (NC). **(B)** Depicts the SPSM of the LM, REST, and AR references at the time range of 210–290 ms.

For the consideration of simplicity, ERP waveforms of four conditions in frontal electrodes (F7, FZ, and F8) and posterior electrodes (PO7, POz, and PO8) were displayed in Figure 3 for the three types of reference (LM, REST, and AR). Based on previous studies and the results of the data-driven analysis, mean amplitudes were averaged at N270 latency (210–270 ms) and posterior P2 latency (210–290 ms) at the frontal electrode clusters (N270 [F1, FZ, F2]) and the left posterior electrode clusters (P2 [PO5, PO7, P7]) for three references separately. The use of the mean value of ROI and multiple adjacent electrodes was to improve the signal-to-noise ratio (Keil et al., 2014) of ERP data. Figure 4 shows the scalp distributions of N270 and posterior P2 within the time window of 210–290 ms in all conditions and for three references separately. Besides, to clearly show the change detection-related ERP effects based on each reference at the time range of 210–290 ms, the scalp distributions of the difference waves were shown in Figure 5A. As can be seen, N270 culminating at the central frontal sites and posterior P2 culminating at the left posterior sites in all types of references. Then, for each type of reference, a one-way repeated-measure ANOVA with the factor of change type was conducted.

**Figure 6:**
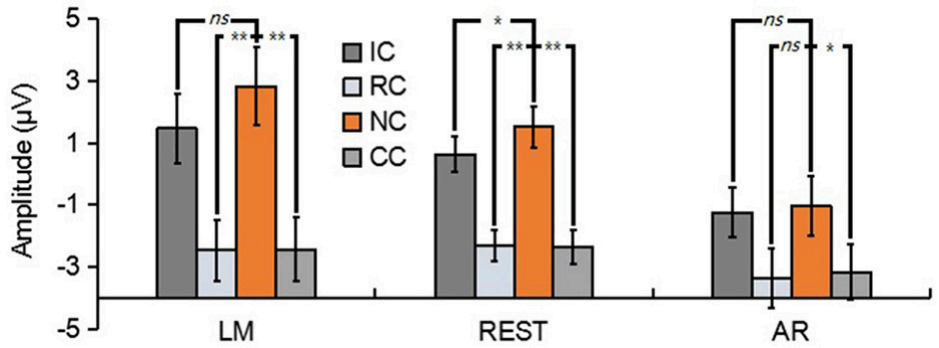
Mean amplitude of the N270 (210–270 ms) of each condition for three types of references by averaging the electrodes at frontal electrode cluster [(F1 + Fz + F2)/3]. ns, non-significant; ^*^p < 0.05; ^**^p < 0.01.

For N270, the results revealed a significant main effect in LM [*F*_(3, 39)_ = 17.86, *p* < 0.01, $\eta_{p}^{2}$ = 0.58], REST [*F*_(3, 39)_ = 23.89, *p* < 0.01, $\eta_{p}^{2}$ = 0.65] and AR [*F*_(3, 39)_ = 6.79, *p* < 0.05, $\eta_{p}^{2}$ = 0.34] (see Figure 6). *Post-hoc* contrast showed that in LM, the N270 amplitude of NC was significantly lower than that of RC (*p* < 0.01) and CC (*p* < 0.01), no significant difference was found between IC and NC (*p* = 0.07); in REST, the N270 amplitude of NC was significantly lower than that of RC (*p* < 0.01), CC (*p* < 0.01) and IC (*p* = 0.02); while in AR, only CC elicited a significantly more negative N270 (*p* < 0.05), but not RC (*p* = 0.13) and IC (*p* = 1).

**Figure 7:**
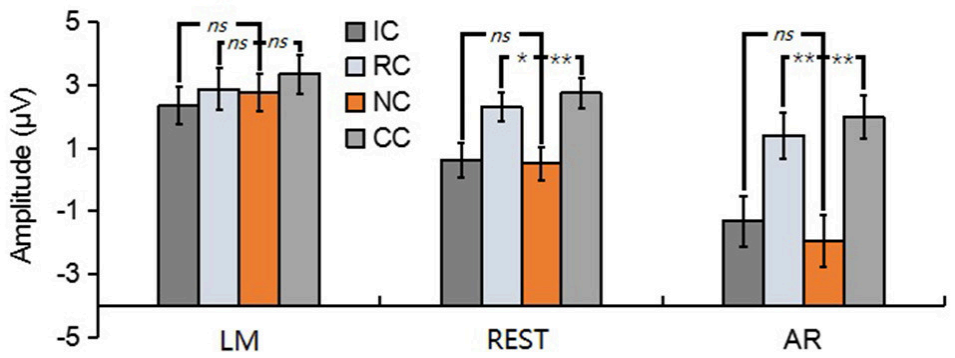
Mean amplitude of the posterior P2 (210–290 ms) of each condition for three types of references by averaging the electrodes at left posterior electrode cluster [(PO5 + PO7 + P7)/3]. ns, non-significant; ^*^p < 0.05; ^**^p < 0.01.

For posterior P2, the results revealed a significant main effect in REST [*F*_(3, 39)_ = 16.62, *p* < 0.01, $\eta_{p}^{2}$ = 0.56] and AR [*F*_(3, 39)_ = 28.77, *p* < 0.01, $\eta_{p}^{2}$ = 0.69], but not in LM [*F*_(3, 39)_ = 1.76, *p* = 0.19] (see Figure 7). *Post-hoc* contrast showed that in REST, the posterior P2 amplitude of NC was significantly lower than that of RC (*p* < 0.05) and CC (*p* < 0.01), no significant difference was found between IC and NC (*p* = 1). In AR, the same pattern of results was obtained. Concretely, the posterior P2 amplitude of NC was significantly lower than that of RC (*p* < 0.01) and CC (*p* < 0.01), no significant difference was found between IC and NC (*p* = 0.20).

### SPSM results

Scalp distributions of *p*-value for the main effect of change type were used to form the statistical parametric scalp mappings (SPSM), which was used to represent the experimental effect of each type of reference. As can be seen from Figure 5B, the SPSM of each reference was different from each other, with AR mainly indicated a left posterior distribution, REST indicated center to right anterior and left posterior distributions, and LM indicated a center to right anterior distribution. Obviously, each type of reference yielded a distinct distribution pattern of the task-related ERP effect.

## Discussion

In current study, the change detection-related ERP effects were systematically investigated by comparing the LM, REST, and AR references in a delayed matching task. To avoid the contamination of distinctive artifact removal standards on the reference effects, re-reference was done after all preprocessing had been completed. As predicted, both of the SPSM and the change detection-related ERP effects were changed by the adopted references. Specifically, the ERP results showed that in LM, both the relevant change and the conjunction change elicited significantly more negative N270, but not the irrelevant change. Notably, the change detection-related posterior P2 effect disappeared under LM. In AR, only the irrelevant feature change elicited a significantly more negative N270, while both the relevant change and the conjunction change elicited significantly more positive posterior P2. In REST, however, all the three types of change conditions elicited significantly more negative N270. Besides, both the relevant change and the conjunction change elicited significantly more positive posterior P2. Consistent with these findings, the SPSM showed a left posterior distribution of task-related ERP effects for AR, an anterior distribution for LM, and both anterior and left posterior distributions for REST.

### Reference effect on the ERP amplitude

An objective fact is that the inverse problem of EEG is reference-free. It means that the spatial distribution pattern of the scalp voltages is not affected by the references used (Geselowitz, 1998; Yao, 2001; Yao et al., 2007). In the current data, we also observed that identical spatial distribution patterns of the scalp voltages of each condition were observed across all three types of references (Figure 4). However, consistent with previous findings (Kayser et al., 2007; Yao et al., 2007; Tian and Yao, 2013; Liu et al., 2015; She et al., 2017), ERP effects in current study also vary depending on the adopted references. In this case, each type of reference could provide distinct results from the same data. Therefore, three distinct possible explanations were suggested: (1) based on LM, the frontal conflict control system was mainly involved in detecting changed information, and the conflicting effect caused by the task irrelevant feature change was effectively ignored; (2) based on AR, it could be the case that the change detection process mainly involved the posterior attention mechanism, and only the detection of conjunction feature change requisitioned the frontal control system; (3) based on REST, both the posterior attention mechanism and frontal conflict control system were involved in the change detection processing, and therefore more in line with the multi-stage theory of percept-memory comparison (Hyun et al., 2009; Yin et al., 2012). Besides, only the REST-obtained N270 showed a significant increment in the task-irrelevant feature change. This finding was compatible with the delayed behavioral performance, and therefore is consistent with the view that frontal conflict control mechanism characterized by N270 is responsible for regulating external behavioral conflicts (Zhang et al., 2008; Yin et al., 2012; Scannella et al., 2016). Obviously, the interpretation of the ERP results varied with the references used, and only the REST provided the closest approximation to the relevant literatures and the delayed behavior performance.

### Reference effect on the SPSM

Previous studies found that SPSM varies depending on the adopted references (Tian and Yao, 2013; Yang et al., 2017). Consistent with these findings, we also observed that SPSM in current study varied with the references used (see Figure 5B). However, only one result is the most reasonable. The distribution of SPSM closest to the real case should correspond to the brain regions activated in the change detection processing. By using a delayed matching task as the current study, Zhang et al. (2008) found that the detection of changed information mainly activated the left occipito-temporal cortex, together with the right ACC and the right DLPFC. Notably, only the SPSM obtained by the REST was consistent with the scalp distribution of activated brain regions of Zhang et al. (2008), but not the other two types of references, thus confirming the superiority of the REST technology.

### Why is rest more reasonable?

**Figure 8:**
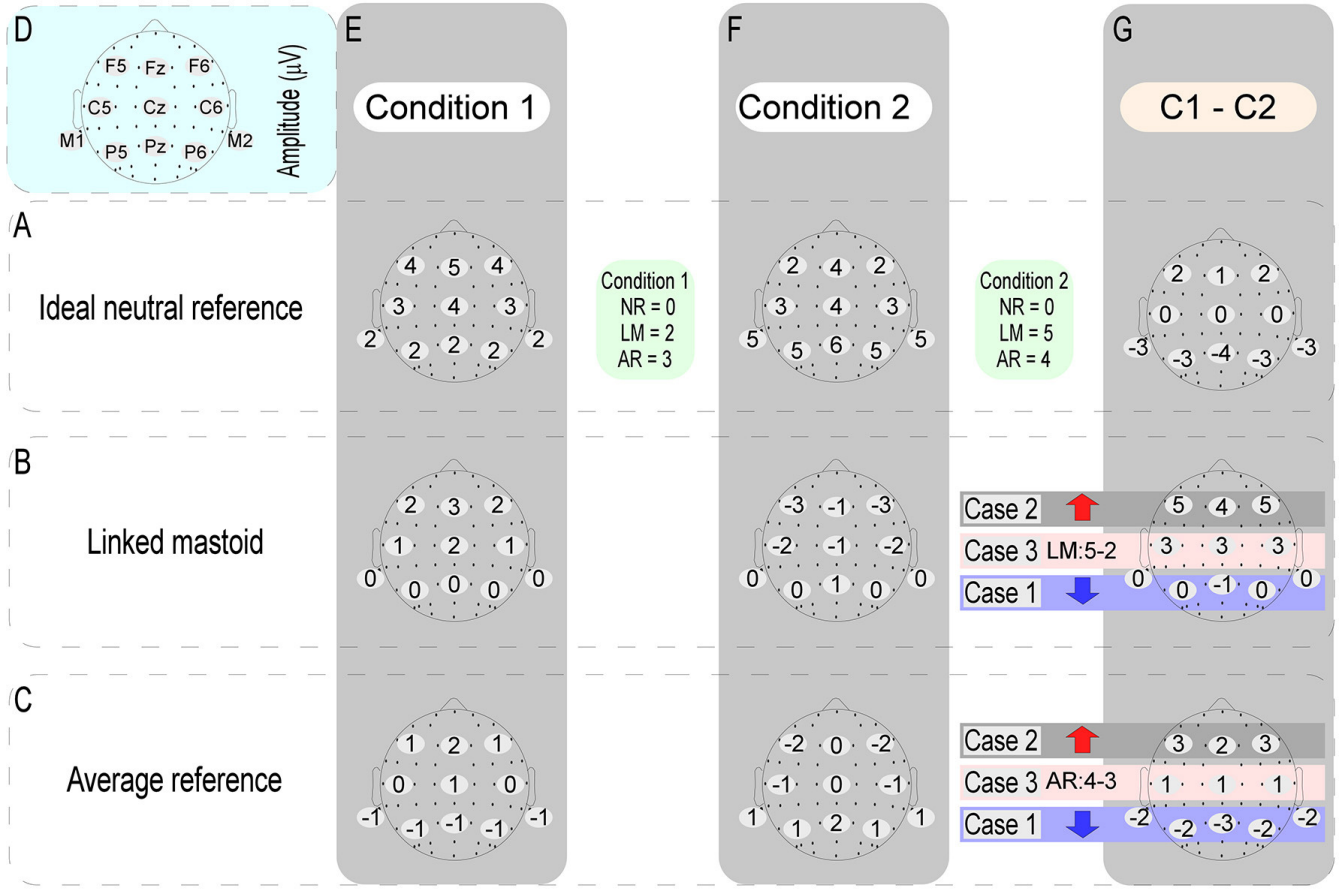
The above figure shows when one try to calculate the significant difference between the ERP amplitudes of two experimental conditions (C1 - C2) at a certain time point, the distortion of the data induced by using of the non-neutral reference [i.e., average reference (AR) and linked mastoid (LM)] might be conveyed in three forms. We took the ERP data obtained by the ideal neutral reference (row A) as the true data. The other two types of references were referenced based on this data. As can be seen from above, compared with the true difference values (the difference map of row A and column G): (case 1) if the voltage values of any one of the electrodes (here, take F5, FZ, and F6 electrodes as examples) between two different experimental conditions have the same changing trend with their respective reference values, then their amplitude difference would be falsely reduced (as indicated by the blue arrows); (case 2) if the voltage values of any one of the electrodes (here, take P5, PZ, and P6 electrodes as examples) between two different experimental conditions have an opposite changing trend with their respective reference values, then their amplitude difference will be falsely increased (as indicated by the red arrow); (case 3) if the voltage values of any one of the electrodes (here, take C5, CZ, and C6 electrodes as examples) between two different experimental conditions are the same, however, the amplitude differences between them will depend entirely on the difference of their respective reference values.

In the ERP study, cognitive neuroscientists tend to pay more attention to the ERP amplitude differences induced by different experimental conditions. Tian and Yao (2013) have been pointed out that the effect of references choice on the wave amplitude of each experimental condition is a constant at the same time point of each electrode, while the constant at different time points is variable. This view explains why the task-related ERP effects in previous studies (Tian and Yao, 2013; She et al., 2017) and the current data varied with the references used, and indicates that the effects of the references choice to EEG data are unavoidable. In this case, if the adopted reference is non-neutral, task-induced electric potentials would be mixed into the calculation of the reference value of each experimental condition. If these mixed neural electric potentials vary depending on the experimental conditions, then the reference value of each experimental condition would not be a constant. This will inevitably distort the true amplitude differences between different experimental conditions. When one try to calculate the significant difference between the ERP amplitude of two experimental conditions at a certain time point, this kind of distortion induced by the use of the non-neutral reference would be manifested in three forms: (1) if the original electric potentials of two experimental conditions have the same changing trend with their respective reference values, then their amplitude difference would be decreased; (2) if the original electric potentials of two experimental conditions have an opposite changing trend with their respective reference values, then their amplitude difference would be increased; (3) however, when the original electric potentials of two experimental conditions are the same, the amplitude difference between them will depend entirely on the difference of their respective reference values (in detail, Figure 8 illustrates a specific case). Take the LM reference as an example. This method assumes that there is no electrical activity at the electrode sites of the bilateral mastoids. However, this assumption does not hold, since there is no genuine electrically inactive reference site on the head (Katznelson, 1981; Nunez et al., 1997). Considering that bilateral mastoids adjoin the temporal occipital regions, the task-related electrical activities in these areas would inevitably be mixed into the calculation of the reference value of LM. This will weaken the electrical signals of the bilateral temporal occipital regions, while the signals away from the bilateral mastoid sites (i.e., the central frontal region) might be falsely increased. This reasoning is consistent with the current results and previous findings (Tian and Yao, 2013), which showed that the ERP power of LM was markedly shifted to the frontal region. Besides, this view can also explain why the change detection-related ERP effects in the left posterior area of current study was erased when the LM was used.

Unlike the LM, AR uses the average of all the scalp electrodes, and thus is regarded as unbiased to any electrode sites. The basic assumption of AR is that the surface potential integral of a volume conductor is zero at any given time point (Geselowitz, 1998). This means that when the whole brain is densely covered by electrodes and close to the sphere in its shape, the average of the potentials recorded at these electrode sites will be infinitely close to the theoretically desired zero value (Geselowitz, 1998). However, this kind of electrode setting is almost impossible to achieve in the real operations, in consideration of the spatial distribution of electrodes being limited to the upper part of the head (i.e., the electrode caps used in the current study). In this case, similar to LM, the reference values of AR for different experimental conditions would not be a constant. Supposing that the amplitude difference between two experimental conditions occurs in a portion of the brain region (i.e., the frontal area of current study and the posterior region of Tian and Yao), in the light of the above logic, the task-related ERP effects in this areas might be attenuated and erroneously transferred to other brain regions (i.e., the frontal region of Tian and Yao and the posterior area of current study) when AR was adopted. Recently, the theoretical shortcomings of AR were also confirmed by Yao (2017). By showing three counter-examples, Yao pointed out that AR is not good in theory in general.

Compared with the other two types of references, REST is not a channel-based reference. More importantly, REST has a solid theoretical support from the mathematical physics (Yao, 2001; Yao et al., 2007). Approximately reconstructing a point far away from all brain sources and scalp electrode sites, REST was proved to be closer to the neutral reference (Yao, 2001; Zhai and Yao, 2004; Liu et al., 2015; Chella et al., 2016). Therefore, REST is considered the most realistic way to restore the EEG signals. The rationality of the REST was also confirmed by a series of simulation studies. These studies all suggested that the reference values provided by the REST technology are closer to the ideal zero or neutral point (Yao, 2001; Zhai and Yao, 2004; Marzetti et al., 2007; Qin et al., 2010; Liu et al., 2015; Chella et al., 2016; Huang et al., 2017; Lei and Liao, 2017). This means that the results provided by the REST are closest to the actual case. Consistent with this view, REST-based ERP results proved to be the most reasonable in the current data and previous studies (Tian and Yao, 2013; She et al., 2017; Yang et al., 2017).

## Conclusion

In summary, the choice of reference is critical to ERP research. Although the spatial distributions of the scalp voltages were consistent between the adopted references, the task-related ERP effects might be changed. Most importantly, the current data and previous studies have consistently confirmed that REST can provide the most reasonable results. On the physical level, we point out the effects of non-neutral references on the significant differences of wave amplitudes between different experimental conditions. Coupled with the support of many theoretical evidences, we recommend future researchers use the REST technique extensively to better understand our brain.

## Author contributions

TL and ZH designed the research, TL collected the data, TL, YL, and QL analyzed the data, and all authors interpreted the data and wrote the manuscript.

### Conflict of interest statement

The authors declare that the research was conducted in the absence of any commercial or financial relationships that could be construed as a potential conflict of interest.
